# Parental Perceptions of Children’s Exposure to Tobacco Smoke and Parental Smoking Behaviour

**DOI:** 10.3390/ijerph17103397

**Published:** 2020-05-13

**Authors:** Vicki Myers, Laura J. Rosen, David M. Zucker, Shoshana Shiloh

**Affiliations:** 1 Department of Health Promotion, School of Public Health, Sackler Faculty of Medicine, Tel Aviv University, Ramat Aviv, Tel Aviv 6997801, Israel; rosenl@tauex.tau.ac.il; 2 Department of Statistics, Hebrew University, Mount Scopus, Jerusalem 9190501, Israel; david.zucker@mail.huji.ac.il; 3 School of Psychological Sciences, Gershon H. Gordon Faculty of Social Sciences, Tel Aviv University, Ramat Aviv, Tel Aviv 6997801, Israel; shoshi@tauex.tau.ac.il

**Keywords:** tobacco smoke exposure, children, smoking behaviour, perceptions, parents

## Abstract

Around 40% of children are exposed to tobacco smoke, increasing their risk of poor health. Previous research has demonstrated misunderstanding among smoking parents regarding children’s exposure. The parental perceptions of exposure (PPE) measure uses visual and textual vignettes to assess awareness of exposure to smoke. The study aimed to determine whether PPE is related to biochemical and reported measures of exposure in children with smoking parents. Families with at least one smoking parent and a child ≤ age 8 were recruited. In total, 82 parents completed the PPE questionnaire, which was assessed on a scale of 1–7 with higher scores denoting a broader perception of exposure. Parents provided a sample of their child’s hair and a self-report of parental smoking habits. Parents who reported smoking away from home had higher PPE ratings than parents who smoke in and around the home (*p* = 0.026), constituting a medium effect size. PPE corresponded with home smoking frequency, with rare or no home exposure associated with higher PPE scores compared to daily or weekly exposure (*p* < 0.001). PPE was not significantly related to hair nicotine but was a significant explanatory factor for home smoking location. PPE was significantly associated with parental smoking behaviour, including location and frequency. High PPE was associated with lower exposure according to parental report. This implies that parental understanding of exposure affects protective behaviour and constitutes a potential target for intervention to help protect children.

## 1. Introduction

An estimated 40% of children are exposed globally to the harmful effects of tobacco smoke in their homes [1]. Interventions to help parents quit smoking have shown some success; however, the majority of parents continue to smoke [2]. Current research has failed to find an effective way of protecting children, and parental reports of exposure are often inaccurate. This failure may be partly due to a fundamental misunderstanding of exposure based on deficiencies in perceptions, including parental perceptions of what constitutes exposure (PPE) [3,4].

While adult smokers have some choice about whether or not to smoke, children who live, play and study in areas frequented by smokers are forcibly exposed to the harms of tobacco smoke exposure (TSE) including second-hand smoke (SHS) and third-hand smoke (THS). Third-hand smoke comprises components of tobacco smoke which remain on surfaces or are absorbed into fabrics after smoking and subsequently re-released into the air [5]. In addition to the well known health risks of TSE—which in children include lower respiratory infections, ear infections, asthma and sudden infant death (SIDS) [6]—children of smoking parents are more likely to become smokers themselves [7,8]. More recently, evidence has been found for long-term cardiovascular risks associated with children’s exposure [9] and increased child mortality [10]. Small children are particularly vulnerable due to their physiology and faster breathing rate, less developed immune system, and the fact that they are in greater contact with surfaces.

Individual perceptions influence health behaviour. Perceived susceptibility and perceived severity of a health threat, as maintained by the Health Belief Model [11], are important predictors of health behaviour [12]. In the context of tobacco exposure, this would relate to parents’ beliefs about how likely one’s child is to be exposed and how severely this may affect his or her health. However, in order to correctly assess susceptibility and severity, parents must understand the nature of the constructs in question, specifically tobacco exposure. Research has shown that people perceive tobacco exposure in different ways and interpret questions about exposure differently [3]. Awareness of exposure is often based on sensory perceptions, which differ between individuals, and between smokers and non-smokers [4]. Heavy and light smokers may also have differing exposure perceptions [4]. Some people consider exposure to occur only when tobacco smoke can be seen or smelled; while others have a much broader perception of what constitutes exposure. The question remains as to whether these exposure perceptions are related to behaviour and specifically to smoking behaviour.

Studies which compared parental report with biomarkers of exposure, or children’s own reports of exposure with their parents’ reports, show that parents tend to under-report their children’s exposure to tobacco smoke [13,14,15]. In an attempt to shed light on both the discrepancies between parental report and biochemical assessment of children’s exposure, and on the reasons for children’s continued exposure despite the well-known health risks [6], the parental perceptions of exposure measure (PPE) was developed, based on qualitative research with smoking parents [3]. Consistent with evidence that pictorial tobacco health warning labels are more effective and believable than text-only warnings [16], the PPE presents images and textual vignettes of adults smoking around children in different everyday circumstances and asks parents to rate the child’s exposure in these hypothetical situations. Following validation of the measure [4], the current study aimed to test the PPE in a group of smoking parents of young children and to examine the relationship between perceptions of exposure and smoking behaviour around children.

Objectives: The aims of the study were to assess the relationship between PPE and children’s exposure measured both by parental report (home smoking location and frequency) and an objective measure (hair nicotine).

## 2. Materials and Methods

This study used data from Project Zero Exposure, a Phase III randomised controlled trial (RCT) conducted with 159 participants (NCT02867241) in 2016–2018, and designed to help parents reduce their children’s exposure to tobacco smoke. Ethical approval was received from the university’s institutional review board and the Assaf Harofeh Hospital Helsinki committee. Parents provided informed consent for their own and their child’s participation in the study and received a gift voucher at the end of the study as a token of thanks for their participation.

Parents of children up to age 8 were recruited via daycare centres and parents’ groups on social media. Inclusion criteria were: having a child up to age 8, at least one parent smokes at least 10 cigarettes per week, willingness to provide children’s hair samples and availability for the 6-month study period.

A total of 159 families were recruited to the RCT and randomised into three groups: intervention (*n* = 69), control (*n* = 70) and enhanced control (*n* = 20). The sample for the current study included 82 families from the intervention and enhanced control groups in which a parent completed the PPE at baseline (a further 7 families were eligible but did not complete the questionnaire, thus were not included in this analysis).

The study data were obtained at home visits made by interviewers. Parents provided information on their smoking habits, showing interviewers where they habitually smoke and reported on their child’s exposure; a hair sample was obtained from one child in each family.

### 2.1. Measures

**Figure 1:**
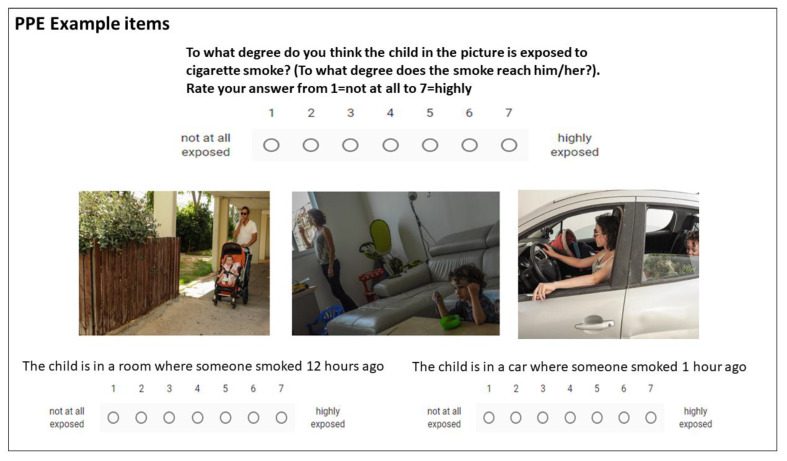
Example parental perceptions of exposure (PPE) items.

Parental Perceptions of Exposure: The PPE is a reliable and validated measure [4], consisting of 23 items: 8 picture items and 9 text vignettes, describing situations of smoking around children in the home, car and outdoors, or specified amounts of time after smoking in the home and car. Six further questions concern perceived knowledge. Participants are asked: “to what extent is the described child exposed to tobacco smoke (to what extent does the smoke reach him/her)?” on a scale of 1 to 7 for each item on the PPE. Item scores were summed to give a total PPE score and divided by the number of items to give a mean score. Higher scores denote a broader definition of exposure and rating children as more exposed in hypothetical situations where tobacco smoke is present. See Figure 1 for example questions. As previously reported [4], a factor analysis of the items produced 6 reliable factors termed: (1) second-hand exposure; (2) third-hand exposure; (3) perceived knowledge/certainty; (4) sensory perceptions; (5) time perceptions; and (6) distance perceptions.

Parentally reported exposure: Participants were interviewed regarding their smoking habits, and the number of cigarettes smoked daily. Smoking status was defined as current daily smoker, current non-daily smoker, ex-smoker or never smoker. Combined parental smoking status was classed as: 0 = none, 1 = mother smokes, 2 = father smokes, 3 = both smoke. Parents provided information on their children’s exposure to tobacco smoke, specifically home smoking frequency (referring to the frequency with which anyone, including guests, smoke in and around the home) and frequency of exposure outside the home (6 points for several times/day, 5 points for once/day, 4 points for several times/week, 3 points for once/week, 2 points for rarely, 1 point for never). Parents also reported where exactly they smoke in the home environment (parental home smoking location) classified into 4 categories: (1) smoking in the whole home; (2) on an inside closed balcony, by a window or designated room; (3) on an outside open balcony; (4) smoking only away from home. Since only 1 family reported smoking in the whole home, categories 1 and 2 were combined as ‘smoking indoors’.

Hair nicotine: All children provided hair samples at the beginning of the study. Hair nicotine was analysed by the Second-hand Smoke Exposure Assessment Lab at Johns Hopkins Bloomberg School of Public Health. Gas chromatography with mass spectrometer detection (GC/MS) was used, and results were given in ng/mg of hair, and whether they were above or below the limit of detection. The LOD was determined by the laboratory for each batch of samples and varied between 0.014 and 0.18 ng/mg. Hair samples were washed prior to analysis, and quality checks were performed. Hair samples of less than 10 mg were considered uncertain, according to the laboratory and were not included in the final analysis.

### 2.2. Analyses

A correlation analysis (Pearson, single correlations) was used to assess the relationship between PPE (mean score and subscales), hair nicotine, number of cigarettes smoked daily by parents and home smoking location and frequency. Independent sample t-tests and an analysis of variance (ANOVA) were applied to analyse the difference in PPE scores according to home smoking location and frequency. For home smoking location, categories were combined into smoking indoors and on balcony vs. smoking away from home. Regression analysis was used to examine how much variance in home smoking behaviour could be explained by perceptions of exposure.

## 3. Results

### 3.1. Sample Characteristics

Of the 82 families completing the PPE questionnaire at baseline, there were 74 participants with hair nicotine data (sufficient hair samples). Mean age of children was 3.3 years (SD = 1.92; range 6 months to 7.7 years). The majority of respondents were educated (63% of mothers and 41% of fathers with an academic degree); working parents (79% of mothers; 90% of fathers); 26% described themselves as of average socioeconomic status (SES), 22% below average and 46% above average (5% declined to answer). Nine per cent described themselves as religious, 26% as traditional, and 65% as secular. Around a third of the hair nicotine samples (35.1%, *n* = 26) were below the limit of detection (LOD) for nicotine, while two thirds (64.9%, *n* = 48) had hair nicotine above the limit of detection. See Table 1 for sample characteristics.

### 3.2. Perceptions and Parentally Reported Exposure: Parental Home Smoking Location

**Figure 2:**
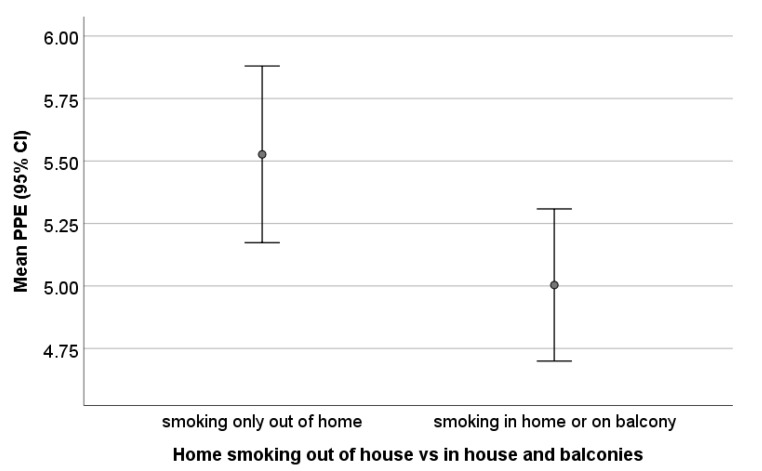
Parental perceptions of exposure according to parental home smoking location.

Regarding home smoking rules, 28 (34.1%) participating families allowed smoking inside the home (including at the window, living room, internal balcony or in a designated room), 32 (39%) usually smoked on an outside balcony (with the door to the home open or closed), and a further 22 (26.8%) smoked only away from the home. Mean PPE score differed according to the home smoking location (see Figure 2): families who reported smoking only outside the home exhibited higher PPE (mean 5.53 ± 0.80, *n* = 0.22) than those who reported smoking in the vicinity of the home including balconies (mean 5.00 ± 1.18, *n* = 60) (*t* = 2.29; *p* = 0.026) (independent samples t test) (Table 2 and Figure 2). The effect size for this difference was d = 0.571.

An additional analysis of PPE subscales showed that parents who smoke in the home or on the balcony consistently gave lower ratings compared to those who smoke only outside the home on all subscales; the difference was significant only on the second-hand smoke factor (*t* = 2.332; *p* = 0.02) (independent t test) which consisted of picture items, while the other factors consisted of text items.

### 3.3. Perceptions & Parentally Reported Exposure: Home Smoking Frequency

PPE corresponded with parental reports of frequency of home exposure (rated on a scale of 1–7), with those reporting rare or no exposure in and around the home having higher PPE scores (5.86 ± 0.44) than those who reported daily or weekly exposure (5.06 ± 1.14) (*p* < 0.001).

### 3.4. Perceptions & Hair Nicotine

Parental perceptions of exposure did not correlate with log hair nicotine (PPE: r = 0.081, *p* = 0.490). Hair nicotine was significantly higher in families where both parents were smokers, compared to just one parent (*p* = 0.016).

### 3.5. Associations between Perceptions, Smoking Characteristics and Demographics

PPE were not significantly correlated with any demographic (child’s age, socioeconomic status, parental education) or smoking factors (number of cigarettes smoked). When broken down into subscales, the perceived knowledge subscale was negatively associated with education, with higher maternal education level related to a lower perceived knowledge score (r = −0.268, *p* = 0.022). The distance sub-scale was related to the total number of cigarettes smoked daily by parents—heavier smokers gave shorter distances that smoke travels outdoors compared to lighter smokers (r = −0.357, *p* = 0.017).

### 3.6. PPE as a Predictor of Home Smoking Location

In a binary logistic regression with parental home smoking location (in home environment including balconies vs away from home) as the dependent variable and PPE, the number of cigarettes smoked daily, child’s age, parental smoking status, SES and home smoking frequency as independent variables, the model was significant, explaining between 20%–30% of variance in home smoking location. PPE was the only significant explanatory factor with an odds ratio of 0.905 (95% CI 0.836–0.979) indicating that for each additional point on the PPE, there were 9.5% lower odds of smoking in and around the home.

## 4. Discussion

Parental perceptions of exposure scores were related to parental smoking behaviour as reported by parents of young children enrolled in a trial, but not to a biochemical marker of children’s exposure. Smoking in the home or on the balcony was associated with reduced perceptions of exposure compared to smoking outside or away from the home environment. Parents who reported more protective behaviour and less smoking around their children tended to higher PPE scores—rating higher exposure scores in hypothetical situations. This was particularly evident with regards to pictures rather than text items, and more closely associated with questions about second-hand than thirdhand exposure.

Home smoking location is very important when considering children’s exposure to tobacco smoke. There is evidence that partial home bans, or selective protection such as smoking in a different room or ventilating the home, are not sufficient to protect children from the harmful effects of smoke (e.g., [17]), though some measures may reduce exposure [18], and that only strict home smoking bans are sufficient to reduce children’s exposure [19,20]. Another study using biomarkers found that children of parents who make an effort to protect their children from exposure to tobacco smoke had 5–7 times higher exposure than non-smoking households, though less exposure than those who smoked indoors and did not take protective measures [21]. These high levels of exposure in children whose parents report taking some protective measures either highlight inaccuracies in parental report, and/or may demonstrate the effects of thirdhand (in addition to second-hand) smoke, whereby residual smoke remains on surfaces, fabrics, furniture, and is later released back into the air and ingested by small children who are particularly vulnerable [5].

Previous research in Taiwan found an association between parental attitudes towards smoking in the presence of children and having a home smoking ban [22]. While they did not measure parents’ ratings of exposure in different circumstances, they did find a relationship between parental attitudes and parental smoking behaviour, regarding questions such as ‘smoking has a bad impact on children’s health’ and ‘only ill children are affected by second-hand smoke’. In a survey of Swedish parents, those who smoked indoors had more positive attitudes to smoking and expressed different ideas about how to protect children than did outdoor smokers, for example, more indoor smokers considered it reasonable to smoke in the kitchen if it is subsequently ventilated [23]. While it is often suggested that in order to protect children, parents must be made more aware of the harm caused to children by smoking, we propose that parents also need to be made aware that exposure is indeed occurring.

The PPE intentionally makes use of photos to assess perceptions. There is evidence that pictures are more likely to be recalled than words [24], particularly regarding health information [25], indeed according to the Dual Process Model of reasoning, images appeal more to the associative, intuitive, system 1 of thinking, which processes data more quickly, while words appeal more to the slower, more deliberate, analytical cognitive system 2 [26,27]. The inclusion of images in the measurement of both exposure perceptions and reported exposure of children could potentially provide more accurate data combining both the appeal of images over text and anchoring the respondent since images are easier to interpret than words. Seeing a photo of real children in a real-world and familiar situation may additionally have more impact than reading about a hypothetical child in the same situation.

Since parental perceptions and reported exposure were assessed concurrently, we cannot determine the direction of the relationship, that is whether perceptions of exposure affect parental smoking behaviour in the home or whether parents’ smoking behaviour affects the way they rate exposure. It is possible that parents who smoke indoors or more frequently around their children give lower exposure ratings as a defensive measure of self-justification or social desirability bias [28]. Among smokers, there is a tendency to ‘optimistic bias’ concerning their own vulnerability to smoking-related disease [29]. Indeed, in a study of smokers and non-smokers, Masiero et al. found a tendency to ‘overestimate the effectiveness of preventive behaviours’ (such as other health behaviours intended to counteract the damage of smoking) in smokers. Further, a recent study demonstrated that most smokers underestimate the relative risk of smoking [30]. It stands to reason that smokers may also underestimate the risks to their own children. Alternatively, or maybe in conjunction, we know that people often rely on their senses to determine whether exposure is occurring and that these are not always accurate or sufficient; indeed regular smokers have a reduced sense of smell [31] which may lead them to give lower ratings of exposure. In our study, heavier smokers gave shorter distances that smoke travels outdoors compared to lighter smokers. These differing perceptions likely affect behaviour and choice of where to smoke outdoors around children.

PPE was not significantly related to hair nicotine levels. Previous literature has shown that there is often a discrepancy between parental report and biomarkers of exposure [13,14]. In the current study, PPE was related to parental smoking habits, including home smoking location. These discrepancies may highlight inaccuracies of parental report or may indicate that reported and biochemical measures assess different things. Indeed parents may misreport their children’s exposure due to social desirability or misunderstanding of the circumstances in which exposure occurs [3]. We attempted to obtain accurate parental reports in the current study by asking detailed questions about where smoking takes place in and around the house. Since data were collected during home visits, parents usually showed the interviewer their usual place of smoking, improving the accuracy of responses. However, children’s exposure may be affected by factors that parents are not aware of or factors beyond parents’ control, for example, exposure at daycare, by grandparents or other caregivers, or in the street, park or playground on a regular basis. In our study, many parents mentioned their own parents being smokers who sometimes smoked around their grandchildren, and some were unsure if smoking occurred in the vicinity of the child’s nursery. Exposure occurring from non-parental sources would be less likely to be related to parental perceptions.

There were some limitations to the study design. Selection bias is inevitable when participants volunteer, limiting the sample to those who were willing to take part in the research. Our sample was largely secular and of average or above SES, with low representation of religious and low SES populations, although participants were recruited from different geographic locations around the country to increase diversity. Participants may have been more aware of the harm of tobacco smoke exposure than non-participants. Hair nicotine has been demonstrated as a valid measure of exposure to tobacco and requires a single sample which shows long-term exposure over the past months (approximately 1cm growth per month) [32,33]. However, one of the limitations of using hair nicotine as our biomarker in this study was that several hair samples were too small (<10 mg) for reliable analysis, and were thus excluded, potentially weakening our results.

## 5. Conclusions

Results from this study show that parents in smoking families have widely varying perceptions of children’s exposure to tobacco smoke and that these perceptions were related to parental smoking behaviour, including location and frequency. Parents generally strive to protect their children but may not be fully aware of the exposure that occurs when they smoke in and around the home environment.

While legislation has helped reduce exposure in public places, young children spend much of their time in the home and are especially vulnerable to the harms of tobacco smoke. Parental smoking behaviour around children is an important and influential part of children’s exposure, and something that can be targeted by intervention. The finding that parental perceptions of exposure are related to home smoking behaviour and reported exposure of children can open up possibilities for targeting these perceptions through intervention, in an attempt to change parental smoking behaviour and reduce tobacco smoke exposure in children’s environments.

## Figures and Tables

**Table 1:** Sample characteristics.

Demographics and Smoking Characteristics: Numerical Variables	*n*	Mean	SD	Minimum	Maximum
Child Age (Years)	80	3.3	1.9	.5	7.7
PPE Mean	82	5.14	1.11	1.65	6.94
Number of Cigarettes Smoked Daily (Mother + Father)	81	14.65	8.76	2	40
Hair Nicotine (ng/mg)	74	0.519	0.897	0.007	6.4
**Demographics and Smoking Characteristics: Categorical Variables**	***n***	**%**			
Mother—Academic Degree	51	62%			
Father—Academic Degree	34	43%			
Parental Home Smoking Location					
Smokes Indoors or on Balcony	60	73.2%			
Smoking Outside and Away from Home Only	22	26.8%			
Home Smoking Frequency (Including Balconies)					
Daily	62	75.6%			
Weekly	11	13.4%			−
Rare or None	9	11.0%			

PPE = parental perceptions of exposure questionnaire.

**Table 2:** Differences in PPE by home smoking location.

Variable	Smoking Only Away from Home (*n* = 22)	Smoking in Home or on Balcony (*n* = 60)	Home Smoking Frequency (Including Balconies)	
			Rare/None	Daily/Weekly
**Mean PPE**	5.527	5.004	5.86	5.06
**SD**	0.797	1.178	0.44	1.14
***p*** **value**	0.026		<0.001	
**Mean Difference**	0.523		0.800	
